# An automatic monitor for lead emissions from stacks: design philosophy and preliminary evaluation

**DOI:** 10.1155/S1463924679000754

**Published:** 1979

**Authors:** C. J. Jackson

**Affiliations:** Health & Safety Executive 403 Edgware Road London NW2 6LN UK


**Table 7. Customer report**

| Chemical data | | | |
|---|---|---|---|
| Vegetation | M1275 | % | 13. 7.79 |
| | Na | K | Ca |
| 1 | 0.017 | 1.4 | 1.4 |
| 2 | 0.011 | 1.3 | 1.5 |
| 3 | 0.015 | 1.4 | 1.4 |
| 4 | 0.055 | 2.7 | 0.68 |
| 5 | 0.073 | 1.8 | 1.2 |
| 6 | 0.064 | 2.0 | 0.79 |
| 7 | 0.011 | 1.1 | 0.31 |
| 8 | 0.021 | 2.6 | 0.69 |
| 9 | 0.026 | 2.3 | 0.71 |
| 10 | 0.45 | 2.4 | 1.6 |
| 11 | 0.45 | 2.6 | 1.5 |
| 12 | 0.45 | 2.7 | 1.2 |
| 13 | 0.015 | 2.0 | 0.61 |
| 14 | 0.021 | 2.1 | 0.59 |
| 15 | 0.009 | 2.2 | 0.84 |
| 16 | 0.10 | 3.6 | 0.56 |
| 17 | 0.10 | 3.0 | 0.51 |
| 18 | 0.10 | 3.2 | 0.38 |
| 19 | 0.013 | 3.1 | 2.1 |
| 20 | 0.017 | 2.6 | 0.53 |
| 21 | 0.009 | 2.8 | 0.65 |
| 22 | 0.008 | 2.7 | 0.68 |
| 23 | 0.035 | 2.1 | 0.58 |
| 24 | 0.061 | 2.0 | 0.42 |
| 25 | 0.029 | 2.2 | 0.43 |

Institute of Terrestrial Ecology
Subdivision of Chemistry and Instrumentation
Merlewood Research Station
Grange-Over-Sands
Cumbria
LA11 6JU

customer report can be produced directly by the system, an example of which is included in Table 7.

Many instruments are now marketed incorporating a dedicated microprocessor. These can be linked into the system described by processing the analogue signals using the instrument microprocessor prior to transfer of the digital readings to the central processing system for further processing, report compilation and archiving.

Despite the relatively small capacity of this data processing system, it has been possible to design a data bank for use in the "stand alone" mode, utilising all of disc 1 for record storage. Input of data via the VDU is quick and efficient and several search programs have been designed to extract data from the discs in the appropriate form. In order to contain as many records as possible on each disc, data has been input as codewords onto disc. Micro-computers will generally run slower than large main-frame computers, as pointed out by Little and Reeves (2), and this should be borne in mind when planning an ambitious data bank utilising a small system. Input of data has to be rigidly standardised in order to minimise this problem.

REFERENCES

[1] Allen, S.E., Grimshaw, H.M., Parkinson, J.A., Quarmby, C., "Chemical Analysis of Ecological Materials", Blackwell.

[2] Littler, J.S. and Reeves, R.M., **Chemistry in Britain.** 1978, 3, 118-126.

### An automatic monitor for lead emissions from stacks: design philosophy and preliminary evaluation*

C.J. Jackson

*Health & Safety Executive, 403 Edgware Road, London NW2 6LN, UK.*

### Introduction

In 1974, the Alkali Inspectorate, at that time part of the Department of the Environment (DOE), concluded that there was a need for a device to continuously monitor metal dust and fume emissions from stacks and ducts. As a first requirement it was decided to investigate the continuous monitoring of emissions from the lead smelting process. Lead and its compounds are major industrial and environmental hazards and emissions of lead fume and dust are monitored against standards set in a Health and Safety Executive Code of Practice for Lead Works.

A contract was, therefore, concluded between DOE and BNF Metals Technology Centre (BNF), for the latter organisation to develop a continuous monitor for lead emissions based on the requirements given in the, at that time, proposed Code of Practice. Indeed, the specific instrument performance was defined as:

(i) To record, reasonably accurately, the level of emission which occurs during normal operation.

(ii) To detect any significant increases in the emissions due to failure of the filter bags in the fume arrestment plant.

(iii) To give a positive meaningful reading within the normal concentration limit of 0.02 g $M^{-3}$.

iv To give a rapid response to concentration up to 0.10g $M^{-3}$.

Finally it was required that such an instrument be designed

* *This paper was originally presented at a Chemical Society, Analytical Division, Automatic Methods Group meeting entitled 'Safety and Automation' at Chester, 5 October 1978.*

for long term, relatively unattended, operation in a works environment.

A variety of suitable analytical procedures are available for the determination of metal fumes and these can involve either the removal of a sample of the fume from the stack or the determination of the fume in situ. The former has the disadvantage of needing to abstract a representative sample from the stock, but has the advantage of using relatively simple and well understood analytical procedures which can be sited away from the stack in a less hostile environment. In addition, one analytical instrument can, in theory, serve a number of different stacks. The latter has the disadvantage of using more sophisticated instrumentation that has to be sited in an exposed position on an individual stack or duct. One advantage is the ability to use a multi-element analytical system. In addition problems associated with sampling and transporting representative samples are avoided.

Having considered these alternative approaches, BNF decided on the former i.e. the remote analysis of a representative stack sample. They therefore evaluated the following analytical techniques: Atomic Emission (AE), Atomic Absorption (AA) and Atomic Fluorescence (AF) Spectroscopy. A decision was made to use an Atomic Fluorescence spectroscopic procedure based on the following conclusions:

(i) AF was preferred to AE because the method of exciting the characteristic lead radiation is specific – thus the risk of unwanted line overlaps from any other elements present in the stack gas is minimised.

(ii) AF was preferred to AA because the lead free signal is zero in AF but is at a maximum in AA – thus AF is less sensitive to short-term variations in the incident radiation intensity; in addition it has a large linear dynamic measuring range.

(iii) In AF, the characteristic fluorescent radiation is emitted equally in all directions. Thus a less restrictive optical layout is possible than for AA. This, together with the use of non-dispersive optics, offers the prospect of a relatively simple extension of the prototype instrument for simultaneous (or sequential) monitoring of several elements, such as Cd, Cu, As, Se, Te etc.

It was appreciated that AF (as with other flame spectroscopic procedures) is best suited to the analysis of fumes (i.e. particules of less than 1$\mu$) – otherwise sensitivity becomes dependent on particle size. Whilst the emissions from stacks fitted with an effective fume arrestment system were not expected to significantly exceed this size limit, this point was investigated and will be discussed later.

### Description of the stack monitor

The continuous stack monitor, as designed and originally constructed by BNF, is illustrated schematically in Figure 1.

Briefly, its operation is as follows: An isokinetic sample is taken from the stack, using a Venturi-type injector, for about 30 seconds every 3 minutes. This is fed, via a heated pipe (to prevent sample drop-out on cooling) to an air-propane flame where it is burnt. The atoms generated in the flame are excited by the 283nm emission from a lead hollow cathode lamp – fluorescence at 405nm being detected using a narrow band-pass filter and a photomultiplier tube. This signal intensity is integrated and compared with a stored calibration signal generated, once every 30 sample measurements, by nebulizing a 1000$\mu$g ml$^{-1}$ lead standard solution and sampling the resultant dried aerosol using the same injector, transport system and analytical procedure as for the samples. The lead level in the stack (in g M$^{-3}$) is fed to a recorder, analogue meter and, if above a pre-set level, to a "high lead" warning circuit giving various alarm indications. Between each sampling period there is a passive or 'nose-blow' mode used for cleaning the probe by diverting the injector airflow back down the probe. Certain fail-safe and warning devices are fitted and the monitor is designed to start up with an initial calibration sequence.

### Probe

An isokinetic sampling probe based on the British Standard, BS 3405, is used to take a representative stack gas sample. It was designed to give a sample flow of 15 1 min$^{-1}$ at a stack gas velocity of 1085M min$^{-1}$.

The probe is constructed from PTFE (enclosed in a stainless steel sheath for protection) and the shape of the nozzle

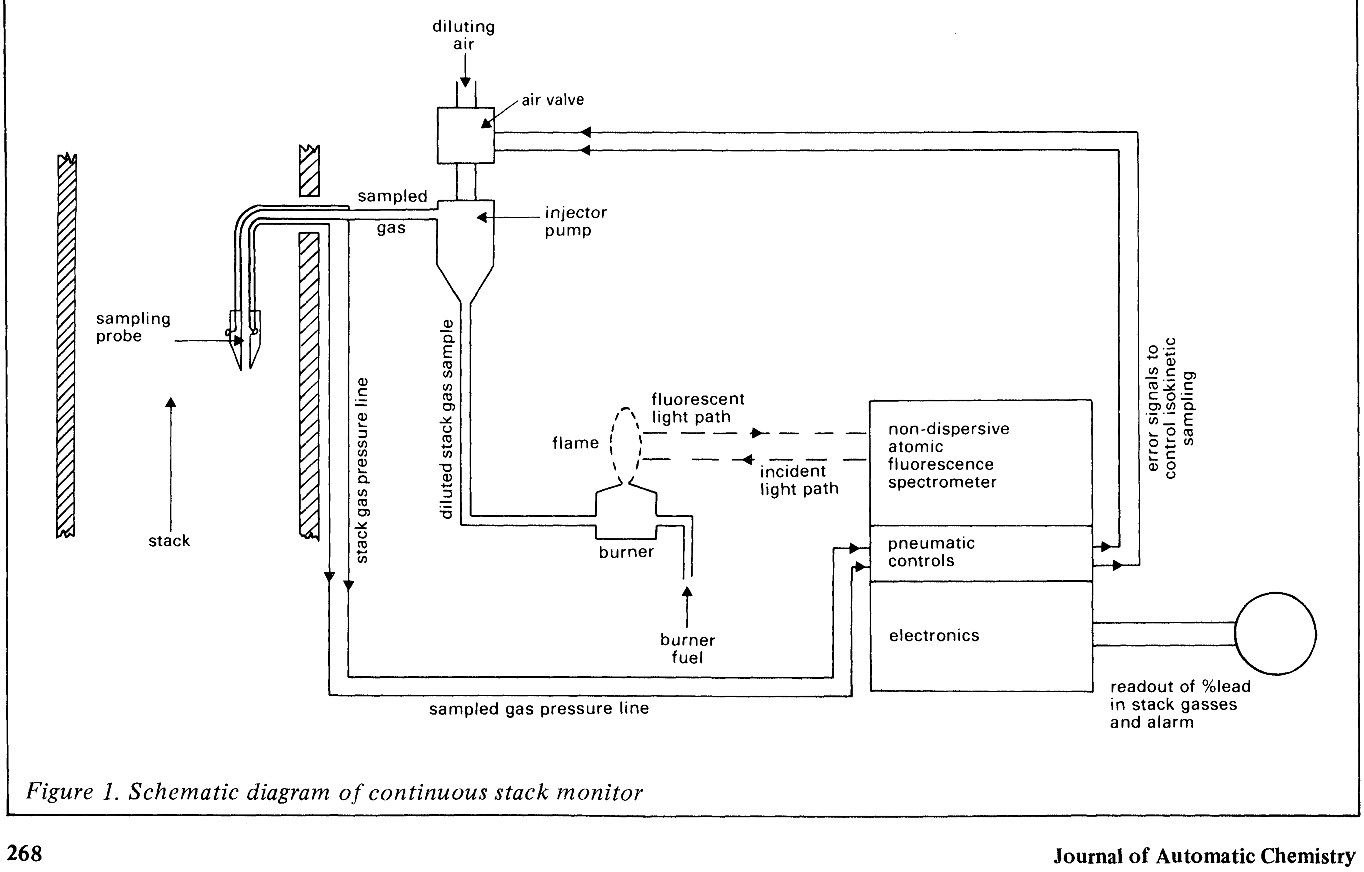

*Figure 1. Schematic diagram of continuous stack monitor*

end was modified from BS 3405 to try to prevent sample drop-out on the tip. To further attempt to keep the probe clean, a 'nose-blow' mode was introduced. The internal and external pressure ports (used to enable the static pressures inside and outside the nozzle to be measured) are connected to an electromanometer situated in the main control unit. This forms part of a feed-back circuit in which pressure differences at the electromanometer are converted into positive or negative electrical signals which control a motorised valve that, in turn, controls the supply of air to the injector; the sample uptake rate is thus adjusted and sampling is maintained in an isokinetic manner.

Mention has already been made that flame analytical procedures are dependent on particle size, especially above 1μ. In addition, the sampling procedure was known to be size selective. BNF, therefore, carried out a series of tests using a Washington sampler to determine the expected particle size distribution. It was shown that under normal operating conditions, the sample particle size range, after the bag filters, is 0-6μ (and mostly 0-3μ) and that of the calibration sample somewhat smaller. It was considered that although there would as a result be some size effects, these would be small and would not invalidate results obtained from the monitor. However, in the event of a bag failure much larger particles would be present in the stack gases. In such a case sampling, and in particular, atomisation in the relatively cool air-propane flame would show a marked size effect and low results would be obtained. It proved possible to show that about a 15% reduction in response (relative to the 1000μg $ml^{-1}$ calibration solution) was obtained when sampling particles of size range 0-8μ. It was agreed that this was acceptable within the defined instrument performance.

### Injector

As with the probe, this is constructed from PTFE, both minimise sample build-up and to prevent attack by the acid stack gases. Further minimisation of sample build-up, both in the injector and in the probe, is achieved by only sampling intermittently and by having a 'nose-blow' period between each sample.

The injector also serves to dilute the stack gas sample to a concentration suitable for direct Atomic Fluorescence measurement. This dilution ratio should be substantially independent of the sample flow rate (which is obviously dependent upon the actual stack gas velocity at any one time) and a 1/16 in diameter orifice was selected giving a 1:5 dilution ratio over a wide range of sample flow rates. To minimise the risks of particulate deposits occurring on the pipework between the sampling probe and burner, it was decided to introduce the sample dilution as soon as possible. To achieve this, the injector was sited in a separate cabinet mounted on the stack being monitored. The diluted sample is then transferred to the burner via about 10m of 19mm i.d. heated pipe.

During calibration the 1000μg $ml^{-1}$ lead solution is nebulized and the resultant aerosol is transported along a second heated pipe and transferred via a branch pipe to the injector. Mechanical plungers (pneumatically actuated) serve to switch the injector inlet to the stack or calibration aerosol as required, and to isolate the calibration system and burner inlet during the 'nose-blow' mode.

### Optical system

The optical layout is shown in Figure 2. Simple non-dispersive optics are used with a Balzer BV6 narrow-band interference filter being used to select the 405.8nm lead fluorescence line. The reference photomultiplier is provided to monitor the output of the lead hollow cathode lamp; the importance of this will be mentioned later.

During early work on the monitor it was noted that the peak transmission of the interference filter drifted by about 0.02nm $^{\circ}C.^{-1}$. Since the band-pass was narrow this caused

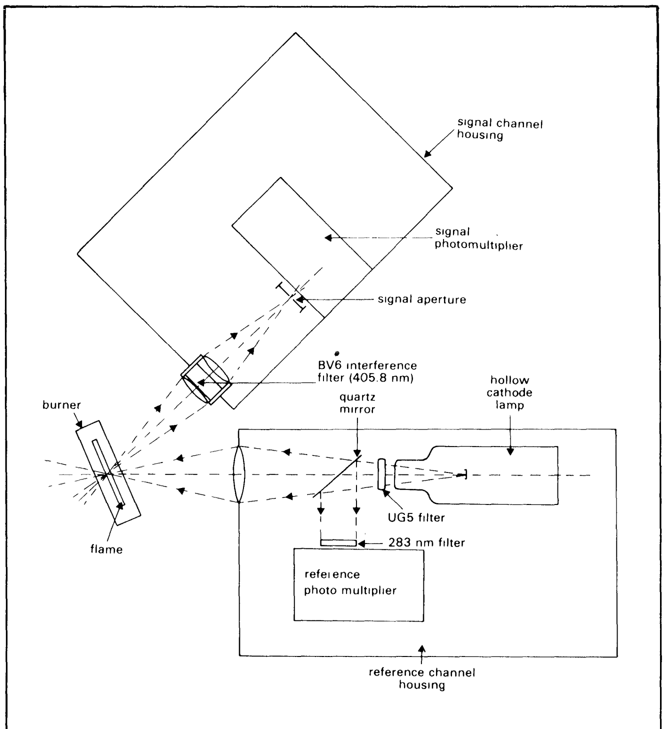

*Figure 2. Optical layout of spectrometer*

severe drift in the overall instrument response. A heating/cooling module was, therefore, installed to control the temperature of the filter and its mounting to 19 ± 1°C. At the same time care was taken to minimise extraneous sources of heat in the optical compartment such as that caused by the air-propane flame. Stray light was also a problem and to provide additional suppression, as well as to eliminate any 405.8nm emission from the hollow cathode lamp, a UG5 filter was inserted in the optical beam; in addition the signal aperture was reduced in size. As a result the signal/noise ratio was doubled with only a 20% reduction in signal intensity.

### Pneumatic control circuits

The pneumatic system, shown in Figure 3, has three operating conditions controlled by two relays R1 and R2 which are, in turn, activated by a sequence controller.

*Passive*

This is a fail-safe mode in which both relays are unenergised, and no power is fed to any of the air solenoid valves. Solenoid valve SV1(P) directs air from the pre-set diaphragm valve DV3 via flow meter F1 to the primary injector in the stack unit. Plunger P1 (actuated by SV5) closes off the air supply to the burner and directs the air flow backwards through the sampling probe (plunger P3 being open) and plunger P2 (actuated by SV3) seals off the branch pipe to the calibration tube. The electromanometer is pneumatically bypassed by SV8 and air is directed backwards through the isokinetic ports via SV6 and DV7.

*Measure*

Relay R1 is energised and power is fed to solenoid valves SV1 (P), SV5, SV6, SV7, SV8 and SV9. Air is fed via the motorised diaphragm valve· DV2 to the primary injector since SV1 (P) is now energised. The isokinetic control system is activated by SV8, SV6 and SV7 enabling the differential pressure at the probe to control the motorized valve DV2 to adjust to a null pressure across the electromanometer. Plunger P1 is opened by SV5 and sampled gas and air from the injector is fed to the burner. The flow into the burner is controlled by the air-mover powered by SV9 via a pre-set valve.

*Calibrate*

Relav R2 is energised and power is fed to solenoid valves SV2, SV3, SV4, SV5 and SV9. Air to the primary injector is supplied via a de-energised SV1 (P) from the pre-set diaphragm valve DV3. Plungers P1 and P2 are open and P3 closed, enabling the injector to sample the calibration aerosol from the nebuliser. Primary air to this is supplied from DV4 via SV2 and flowmeter F2, secondary air coming again from DV4 through a valve and F3. The electromanometer is again pneumatically bypassed by SV8 and air is back-blown into the probe via SV6 and SV7. Sample air to the burner is controlled by the air-mover DV3. via SV9.

*Propane supply to burner*

This is supplied via SV10, which is controlled by the 'flame-out' alarm system, through a pre-set valve and flowmeter F4. Since the flame is momentarily extinguished at each condition changeover, a small pilot flame fed via a pre-set valve is used to re-ignite. In the event of a power failure the main propane supply to the burner is cut off, the pilot flame continues to burn and is sufficient in the absence of a forced draught to reactivate the 'flame-out' sensor when power is restored.

**Electronic circuits**

A block diagram of the electronic system is shown in Figure 4.

*Pulsed hollow cathode lamp supply*

This supplies a pulsed constant current of up to 250mA, at a voltage of up to 350V. The lamp current is controlled by the timing generator to give a burst of 100 pulses of 1.5ms duration, approximately 70mA amplitude, spaced 100ms apart. These bursts recur at intervals of 200s.

*Analogue signal processing*

The signal from the measurement photomultiplier, after amplification, is divided into two channels, one of which is inverted. Channel B is sampled only when the hollow cathode lamp is on. It thus monitors the fluorescent signal plus the background noise. The inverted channel, A, is sampled immediately after the lamp current pulse and so monitors background noise. The two channels are fed to an integrator, the output of which is the fluorescent signal after correction for background. To compensate for changes in the system gain, a calibration signal is derived by nebulizing a standard lead solution and introducing this at the injector, as already described. This calibration process occurs after every 30th stack measurement and is carried out during the interval between measurements. To improve calibration accuracy four groups of 100 pulses are used and the average taken. This average output is sampled and stored digitally. During normal measurement periods, the output of the integrator is divided by this stored calibration signal in a divider circuit. On completion of each measurement period, the divider output is sampled, and held until the next measurement period starts. The 'sample and hold' output drives a scaling amplifier which has a recorder and meter as its outputs.

*Digital sample and hold*

Since the calibration signal needs to be stored for about 90 minutes, digital storage is used. An i/c 8-bit A to D convertor is used; this is enabled at the end of each calibration period and the new calibration value latched in. An i/c D to A convertor continuously reads the output latches, and this signal after scaling is used as the divider denominator.

*Sequence control*

The sequence control provides all the timing pulses necessary for the operation of the electronic circuits and for operating the relays which control the pneumatic sampling system. It is based on a master oscillator and counter system and uses TTL logic, and the sequences already mentioned are shown in Figure 5.

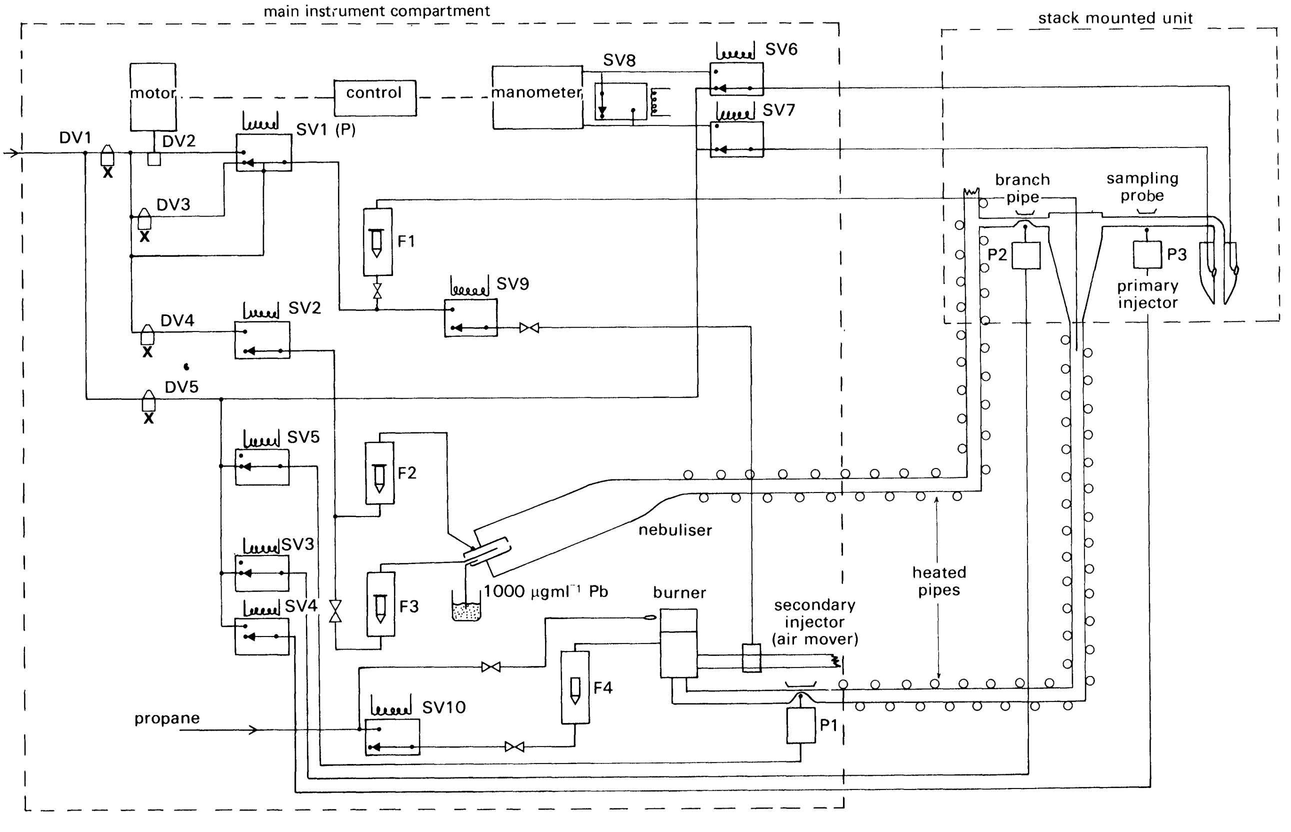

*Figure 3. Schematic diagram of sampling system and associated pneumatics (show in 'no power' condition)*

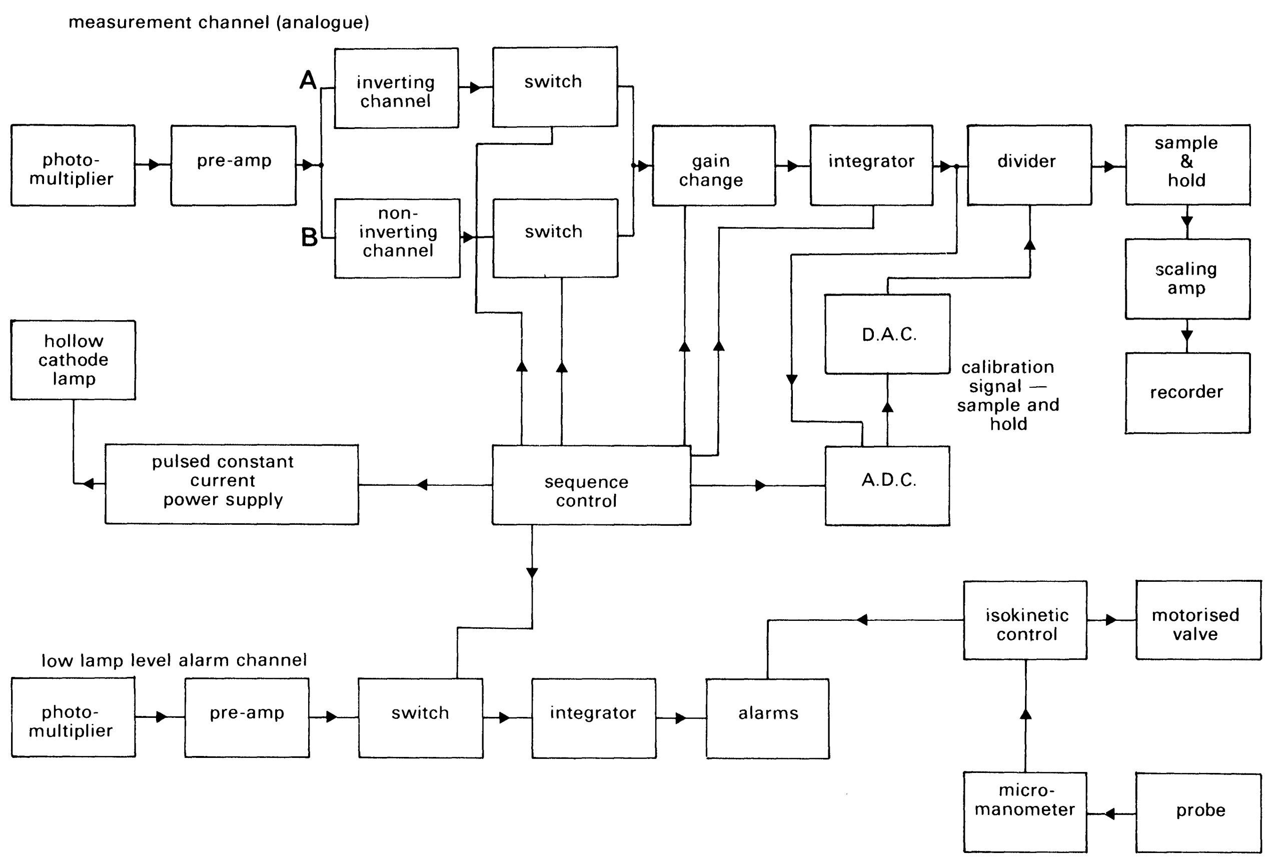

*Figure 4. Block diagram of stack monitor electronic circuits*

*Alarm circuits*

A number of alarm circuits are provided to monitor the performance of the instrument. A malfunction is indicated by the lighting of an L.E.D. for each particular fault. The function alarms consist of low hollow cathode emission, low calibration signal and flame out. The last condition shuts off the propane gas supply to the burner. In addition to the function alarms, there is a high lead alarm which gives a visual indication if the lead level exceeds a pre-set value.

*Isokinetic flow control*

The electromanometer used to measure the pressure difference inside and outside the sampling probe has a sensitivity of ± 1mm WG. TTL logic is used to determine the magnitude and direction of this output and to drive the motorised valve until the pressure differential is zeroed. 30 seconds are allowed for these corrections to be made before the measurement pulses commence (see Figure 5).

### Evaluation of the prototype monitor

The prototype monitor was installed by BNF at a baghouse serving a lead blast furnace. The main console and remote recorder were positioned on the upper level of the baghouse structure, whilst the stack unit was mounted at the N.E. sampling point on the stack. Over a period of 8 days the instrument was run continually without any interruptions. Table 1 indicates the overall stability of the calibration during that period, based on a calibration level of 0.02 g $M^{-3}$ lead. This level of performance was deemed to be adequate.

During this trial period a number of conventional isokinetic samples were taken from the stack and analysed by normal procedures. A comparison of these results with the lead particulate concentration indicated by the continuous stack monitor is given in Table 2. Accepting that the reproducibility

**Table 1 Stability of calibration during long-term works trial**

| Day | Standard Deviation (mg $M^{-3}$Pb) | Relative Standard Deviation (%) | Number of Individual Results |
|---|---|---|---|
| 1 | .98 | 4.9 | 15 |
| 2 | .64 | 3.2 | 14 |
| 3 | .74 | 3.7 | 15 |
| 4 | 1.26 | 6.3 | 14 |
| 5 | .72 | 3.6 | 14 |
| 6 | .78 | 3.9 | 14 |
| 7 | .98 | 4.9 | 14 |
| 8 | .58 | 2.9 | 12 |
| Overall (8 days) | 1.0 | 5.0 | 112 |

**Table 2 Correlation between lead concentrations determined by conventional analytical procedures and those indicated by continuous stack monitor.**

| Determination | Lead Concentration in Stack | |
|---|---|---|
| | Conventional Method (mg $M^{-3}$) | Stack Monitor** (mg $M^{-3}$) |
| 1 | 2.0 | 1.8 |
| 2 | 2.6 | 1.7 |
| 3 | 1.8 | 2.0 |
| 4 | 1.3 | 2.6 |
| 5 | 1.3 | 2.6 |
| 6 | 1.2 | 2.0 |
| 7 | 1.0 | 1.3 |
| 8* | 4.5 | 11.0 |
| 9 | 1.6 | 2.8 |

* *Simulated bag failure, 5 cm slit in bag*

** *Mean value corresponding to period during which conventional sample was taken*

of successive stack samples obtained by conventional measurements is seldom better than ± 30% then four of the nine comparative measurements show acceptable agreement. For the remainder, the levels indicated by the monitor were unacceptably high. At this stage, the OMHL of HSE undertook further development of the prototype instrument, with the aim of overcoming the inadequate reproducibility and other control problems. They then tested on site for a further period of some 8 months. During this period many problems were experienced. Some of these are worth mentioning as they help to illustrate the gulf between having a unit working on a laboratory bench and having the same unit working 24 hours a day in an industrial environment, being evaluated by staff based some 20 miles away.

### Site problems

(i) The stack and associated baghouse were shut down on several occasions with a total downtime in excess of three months.

(ii) The main oil filter on the factory compressed air supply failed and the one installed on the monitor proved to be inadequate and the whole of the pneumatics of the monitor filled with oil before action was taken. The whole unit need stripping down on-site and cleaning.

(iii) Any form of maintenance work high up in the roof of a noisy baghouse is time-consuming and difficult. It is, therefore, imperative that the design is sound and that reliable components are used in the construction of the monitor.

### Design and construction problems

(i) Pneumatic and electronic circuits had been intermixed on the chassis. This clearly causes problems when modifications are needed or cleaning is necessary.

(ii) Sufficient care had not been taken to isolate electronic circuits from the dirty ambient atmosphere.

As a result of these continuing problems, particularly associated with oil in the pneumatics, the monitor was removed from site and returned to the HSE Laboratories for a complete strip down, clean and partial rebuild. The pneumatic and electrical circuits have been separated and the whole of the electronic circuits have been checked and modified, where necessary, to try to minimise failures and to eliminate several problems associated with drift in the pre-amplifiers. The monitor was then retested in the laboratory, and Table 3 shows the performance with a glass reflector plate installed in place of the flame. 11 complete cycles of calibrate followed by 30 measurements are reported.

Further evaluation was carried out by temporarily modifying the monitor to accept a nebulized lead solution as a sample. Table 4 shows the meter output (in arbitrary units), after calibration with a 1000μg ml$^{-1}$ lead solution, when different strength lead solutions were aspirated as pseudo-samples. This performance was considered acceptable. A full test rig was then set up to simulate a stack and thus test out the whole of the monitor under closely controlled conditions before attempting re-installation in a lead works.

Final results are not yet available but it is apparent that there are a number of problems present in the sampling and calibration procedures. It is considered that the Venturi-type injector as designed is likely to *produce* sample drop-out. It is also now known that, under the ambient conditions often experienced on site (wind, rain, cold), the lead aerosol is not being dried properly. This results in calibrant drop-out both in the injector and in the heated tube. The full implications of this are not yet apparent but it seems that the resultant low calibration would produce the high results obtained by the monitor on the original site trials. A new injector has been designed and a calibration procedure proposed which would utilize the 405.8nm emission line from the hollow cathode lead lamp. This signal would be initially calibrated by taking separate stack samples, analysing

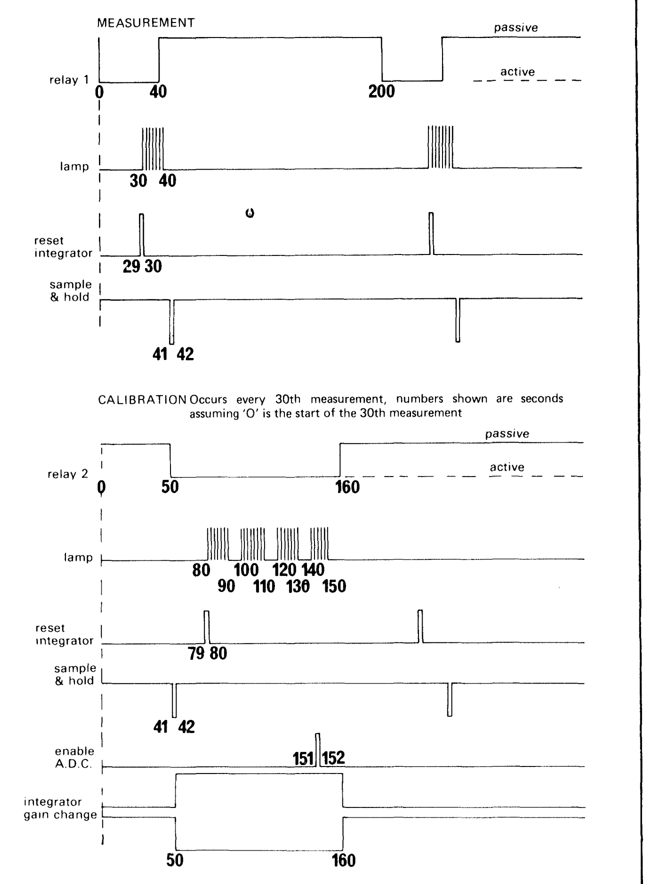

*Figure 5. Sequence control waveforms*

**Table 3 Signal integrator outputs from glass reflector plate**

| Set | Calibration Values (volts) | Mean Measurement Values of each Data Set (30 measurements) (volts) |
|---|---|---|
| 1 | 1.53 | 1.43 |
| 2 | 1.43 | 1.35 |
| 3 | 1.38 | 1.33 |
| 4 | 1.36 | 1.34 |
| 5 | 1.40 | 1.35 |
| 6 | 1.38 | 1.35 |
| 7 | 1.42 | 1.37 |
| 8 | 1.42 | 1.36 |
| 9 | 1.40 | 1.39 |
| 10 | 1.42 | 1.39 |
| 11 | 1.44 | 1.39 |
| Mean | 1.42 | 1.368 |
| Std. Dev. | 0.0448 | 0.0293 |
| Rel. Std. Dev. | 3.1% | 2.14% |

**Table 4 Analogue meter readings, after calibration with 1000μg ml$^{-1}$ lead solution, using simulated samples**

| Psuedo-Sample Concentration (μg ml$^{-1}$ Pb) | 1000 | 500 | 250 | 1000* |
|---|---|---|---|---|
| | 22 | 16 | 5.5 | 25 |
| | 25 | 11.5 | 6.5 | 22.5 |
| | 27 | 10.0 | 6.0 | 24 |
| | 23 | | | |
| Average | 24.4 | 12.5 | 6.0 | 23.8 |

* *Repeat series of determinations*

them and then using these results to set the corresponding monitor readings at the correct level. Once locked-in in this way changes would only be needed if the stack conditions were altered or some significant change made in the sampling procedure.

### Conclusion

The design and evaluation of an automated instrument for the continuous monitoring of lead emissions from stacks is described. This is one of a number of such monitors being designed and evaluated for the Alkali Inspectorate in their search for a reliable, economical instrument for the continuous monitoring of emissions of metal dust and fume from stacks and ducts. The basic concept of the described instrument is seen to be valid but certain problems have been discovered during the evaluation stage. Until the modifications discussed above have been made, and the monitor retested on-site, it is not possible to say that a proven instrument, based on the concepts described, is currently available.

### An evaluation of the Kodak Ektachem system for the determination of glucose and urea *

**R. Haeckel** and **O. Sonntag**
Technical Assistant: **K. Petry**

Institut fur Chemie, Medizinische Hochschule, Hannover, West Germany.

### Introduction

The Kodak Ektachem analytical system is a unique concept recently introduced into the field of clinical chemistry, which applies the company's considerable experience in photography. In contrast to conventional clinical analysis, the chemistry is carried out on a thin film of interacting chemical layers termed a slide. In the analytical instrument produced by Kodak, a dispenser places a drop of serum onto the slide which is then moved into an incubator to develop the colour reaction. The measurements are carried out by reflectance densitometry.

### Thin film chemistry

The principles of this technique can be described by reference to the analysis of urea. Figure 1 shows a schematic diagram of the slide used and also illustrates the chemistry integrated into the multilayers. The spreading layer is an isotropically porous non-fibrous layer with an 80% void volume and a mean pore size of 1.5 microns. The spreading and metering action which is aided by surfactants, compensates for any differences in sample size (nominally 10$\mu$l are applied) and serum viscosity. A constant volume per unit area is then naturally applied to subsequent layers of the slide. High molecular weight materials, such as protein, are removed by this layer and consequently do not interfere with the subsequent analysis. Titanium dioxide is incorporated in the spreading layer to improve its reflectivity.

It also acts as a white background for reflectance measurements against which the colour density produced in the indicator layer can be measured. The reagent layer contains the urease which catalyzes the hydrolysis of urea in the sample to produce ammonia. Water from the serum sample applied, swells the gel allowing the urea to diffuse into the layer. The layer is buffered to pH 7.8 and this maintains the ammonia at a low level, and subsequently extends the range of the assay. A third layer consisting of cellulose acetate butyrate with selective permeability, allows non-ionic materials such as ammonia and water to pass through to the indicator layer. Ionic compounds are excluded, providing some degree of selectivity. The indicator layer consists of a gel-binder incorporating the indicator reagent, in this example, N - propyl - 4 - ( 2, 6 - dinitro- 4 - chlorobenzyl ) - quinolinium ethane sulphonate. Free ammonia which diffuses into this layer reacts with the indicator to form a dye which has a molar absorptivity of approximately 5000 and a broad absorption peak at 520 nm. The reflectance density is measured off the peak at 670 nm. The final layer is a clear polyester support upon which all the other layers are coated. It is transparent and allows measurement of the dye density formed in the indicator layer. Slides for urea and glucose determination are also available. Research has been strongly stimulated by the knowledge that Kodak had developed the multilayer film technique and many more methods are forthcoming.

The authors' experiences with the technique for the determination of the concentration of glucose and urea in serum are presented in this paper. The experimental work was carried out using an investigational unit over a four month period. The evaluation was directed towards assessing the analytical reliability of the thin film concept rather than to the technical reliability and practicality of the instrument used.

In the technique described in detail elsewhere [1, 2], drops of serum are directly applied to the analytical slides. Relatively high concentrations of the analytes to be determined, and also any interferences in the undiluted sample, are therefore in contact with the reagents held in the individual layers. In conventional techniques, samples are usually diluted by factors of 1:10 or 1:1000. Special attention in this evaluation was focussed on determining any possible interferences due to various exogenic and endogenic compounds present in the serum samples.

* Translated from the German by R. Arndt and P.B. Stockwell. The German text of this paper will be published in part in the November 1979 issue of G-I-T Labormedizin.

